# In-Home Video Telehealth for Caregivers and Veterans With Dementia

**DOI:** 10.1093/geroni/igaa057.2285

**Published:** 2020-12-16

**Authors:** Megan Gately, Lauren Moo

**Affiliations:** Bedford VA Medical Center, Bedford, Massachusetts, United States

## Abstract

Home Video Telehealth offers a unique opportunity to support already burdened caregivers of persons with dementia. Veterans Health Administration, through the MISSION Act, is increasingly using video telehealth to provide “care at the right time and in the right place.” Little is known about the benefits and challenges of using video telehealth for in-home caregiver support. We present findings from our seven years offering in-home dementia management to caregivers of Veterans with dementia, that includes supporting caregivers through supportive listening, tips for communication and safety strategies, and recommendations regarding non-pharmacologic management of behaviors. Perceived benefits of in-home video telehealth include an ‘in vivo’ perspective of the family’s natural context and remediating barriers to care such as decreased mobility. Perceived challenges include dealing with technology and privacy concerns. By describing considerations for in-home video telehealth to a clinical population with unique care needs, we inform broader application of a promising technology.

